# Optimizing the Composition Design of Cement-Based Expanded-Polystyrene (EPS) Exterior Wall Based on Thermal Insulation and Flame Retardance

**DOI:** 10.3390/polym14235229

**Published:** 2022-12-01

**Authors:** Jicun Shi, Lei Zhao, Yao Zhang, Hongxing Han, Lihuang Zhou, Chenxi Wang

**Affiliations:** 1 School of Civil Engineering and Architecture, Xinxiang University, Xinxiang 453003, China; 2 Henan Xinsheng Building Energy Saving Decoration Co., Ltd., Xinxiang 453002, China

**Keywords:** external thermal insulation composite, expanded polystyrene (EPS), thermal conductivity, flame retardance, mechanical characterization

## Abstract

The use of thermal insulated decorative panel materials with low thermal conductivity and high flame retardance is a key step toward energy-saving buildings. However, traditional thermal insulation materials are always highly conductive and inflammable, which restricts their application for new buildings. This study aims to prepare the non-combustible, cement-based EPS mixtures with thermal conductivity lower than 0.045 and density less than 140 kg/m^3^ and characterize it with mechanical, thermal, and flame retardant properties. The effect of particle size, Silica coated and content of EPS on the physical, mechanical, thermal, and combustion performance are conducted in this paper. The comprehensive indoor tests including density, water absorbing, softening coefficient, compressive strength, tensile strength, moisture susceptibility, thermal conductivity, and scanning electron microscopy (SEM) along with combustion performance are reported to evaluate the effects of several variables on the investigated cement-based nonflammable EPS (CEPS)mixtures. The results show that small and gradation EPS particles significantly improve the comprehensive performance of mixtures. In addition, Silica coated ESP significantly improve the flame retardance of mixtures while reduce the mechanical characteristics slightly. These results contribute to the selection of appropriate materials to enhance the thermal insulation, flame retardance and mechanical properties of CEPS.

## 1. Introduction

Exterior wall insulation materials should be built with sufficient strength, thermal insulation, and heat insulation performance, to achieve building energy conservation and socially sustainable development [1]. Conventionally, two types of materials have been commonly used, those being organic and inorganic [2]. The organic materials are represented by EPS and extruded polystyrene plate (XPS), while typical representatives of inorganic materials are rock wool board and perlite [3]. However, both materials have some limitation. Lightweight EPS and XPS provide excellent thermal insulation performance, but it is flammable [4]. The EPS and XPS have low strength and are prone to aging, resulting in poor stability and durability [5,6]. In addition, the synthesizing organic materials is high-costed and difficult to recycle [7]. The inorganic materials with stable performance are fire-proof and flame retardant, but with poor thermal insulation performance, and its thermal conductivity is difficult to lower than 0.06. In the past 30 years, rock wool had been used in large quantities [8]. After absorbing water, its bulk density increases, and its thermal insulation characteristic deteriorates rapidly [9]. Worse more, it is unfriendly to construction personnel, so it has been listed as a prohibited material by the management department.

Recently, inflammable insulation board made of inorganic materials composite polystyrene, known as cement-based EPS exterior materials has gained increasing interest as an alternative to the conventional materials [10,11]. Since it is compressed by the mixture of EPS and cement-based materials, it has the advantages of completely bypassing the flammable and thermal insulation concerns [12]. In order to investigate the characteristic of the composite thermal insulation materials, various studies have been conducted [13]. Many studies on the relationship among the thermal conductivity the density, and the EPS content of EPS concrete showed that the thermal conductivity was in line with content [14,15,16]. Wang used cone calibration and butane torch flame burning tests to study the flame retardancy of EPS concrete coated with nanomaterials, the peak heat release rate (HRR), the total heat release (THR), and the total smoke release (TMR) of the composites [17]. In addition, some other studies raised the concerns on the potential effects of various factors, such as material composition, mixture ratio, and impact of additives on the comprehensive performance the mixture [18,19].

The previous studies on the EPS mixture with the density of more than 300 kg/m^3^ focused on the fire resistance and its thermal conductivity which is generally greater than 0.06; meanwhile, the studies on the density less than 200 kg/m^3^ focus on the thermal insulation performance [20,21]. To this end, this study aims to prepare the non-combustible cement-based EPS mixtures with thermal conductivity lower than 0.045 and density lower than 140 kg/m^3^. In order to achieve this objective, comprehensive laboratory tests, thermal conductivity tests, and combustion performance tests were conducted to evaluate the effects of several variables on the CEPS samples. In addition, the non-combustibility test was performed to investigate the fracture surface characteristics after flame. The microscopic features of the CEPS interface bonding characteristics were detected by scanning electron microscopy (SEM). This paper is expected to reveal the characteristic mechanism of CEPS exterior wall insulation material.

## 2. Experimental Program

### 2.1. Materials and Sample Preparation

In this study, the EPS with granules of 2–3 mm and 3–4 mm in size (mean diameter) and a density of 5 kg/m^3^ was provided by Hunan Yue yang Baling Petrochemical Co., Ltd. (Yueyang, Hunan province, China). The cementing materials with Portland cement P.O. of 42.5–80% by weight, was provided by Henan Xin sheng building energy saving decoration Co., Ltd. (Xinxiang, Henan province, China). The ratio of water to cementing materials (W/C) was kept 0.45 consistently for all of the CEPS mixtures. Tap water was used to mix the mixture. Seven CEPS mixtures were investigated in this paper. The control CEPS was made by EPS with particle size 3 mm–4 mm, cementing materials 70 kg/m^3^, and water to cementing materials (W/C) ratio 0.45. The other six EPS with different particle sizes, different contents of cementing materials, and mixing coated EPS with cementitious materials were designed to study the comprehensive properties. Table 1 provides the detailed description of the prepared CEPS. The material parameters in Table 1 are from manufacturer’s recommendations and preliminary test results. The Coated-EPS-CEPS in Table 1 was first adhered to the surface of the EPS using epoxy resin and then mixed with the slurry.

**Figure 1:**
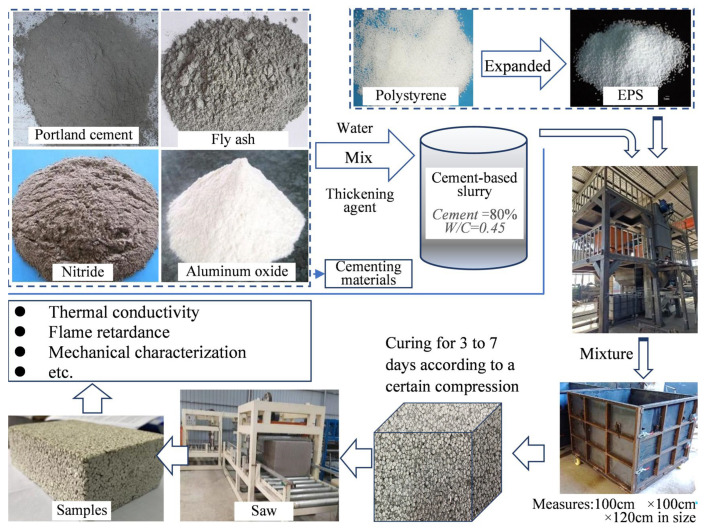
The preparation of CEPS specimen process and test flow diagram.

Figure 1 shows the preparation process and test items of the CEPS. First, the Portland cement, fly ash, nitride, and aluminum oxide were used as the main raw materials with a composite binder to produce a cement-based slurry. Second, a special equipment was used to mix the EPS and slurry evenly, and then the specimens were made at the fixed compression ratio (generally 40%), and maintained at ambient temperature for 3 days to 7 days. Finally, the specimens were sawn to external wall insulation panels at a given size. The thickening agent in Figure 1 was mainly composed of sodium ethoxylated alkyl sulfide and sodium dodecyl sulfonate.

### 2.2. Testing Program

#### 2.2.1. Physical and Mechanical Tests

Physical property tests conducted in this study included density, water absorbing and softening coefficient. The density test was performed based on the Chinese specification JG/T 536 test procedure. For each test, three replicates were prepared.

**Figure 2:**
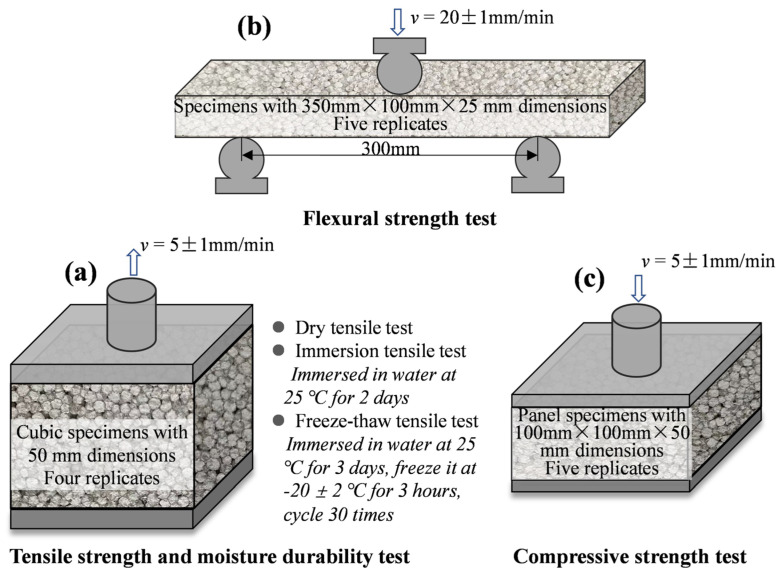
The mechanical property and durability tests: (**a**) flexural strength test, (**b**) tensile strength and moisture durability test, and (**c**) compressive strength test.

The mechanical properties of CEPS were evaluated by three tests, including compressive strength test, immersion tensile strength and freeze-thaw strength test, and flexural strength test [5], as shown in Figure 2.

The compressive strength test was conducted in accordance with GB/T 5486. The compression specimen with size of 100 mm × 100 mm × 50 mm, were compressed at the deformation rate of 10%/min of specimen thickness. The peak compressive stress was taken as the compressive strength value, which shall not be less than 0.15 MPa.

The standard specification JG/T 287 was referred to for testing the tensile strength and moisture susceptibility of CEPS. The specimen with a side length of 50 mm was pasted onto the metal plate with high-strength resin adhesive, and the specimen was stretched to the strength at the time of destruction at the speed of 5 ± 1 mm/min [18]. A dry-wet tensile strength test, and freeze-thaw tensile strength test were conducted to comprehensively evaluate the moisture characteristics. After immersing the sample in water at 25 °C for 2 days, the surface was wiped and tested using the dry tensile strength test method. Similarly, after immersing the sample in water for 3 days, the sample was frozen at −20 ± 2 °C for 3 h, and then the surface of the sample was wiped for tensile strength test. Four replicates were prepared and tested.

#### 2.2.2. Thermal Conductivity

**Figure 3:**
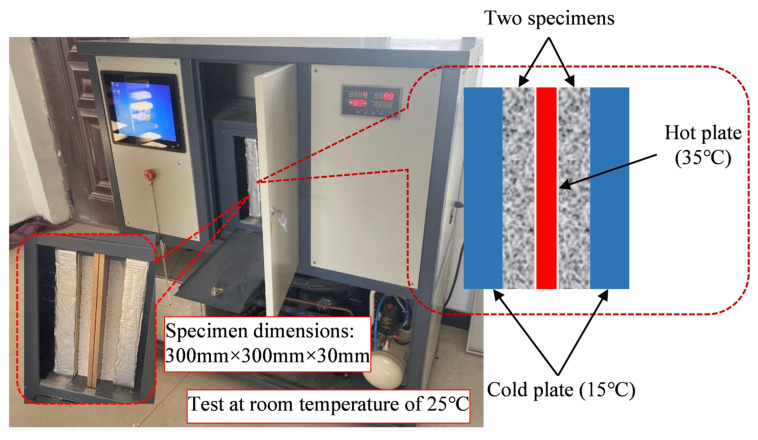
The instrument and specimens for thermal conductivity test.

The thermal conductivity of CEPS was measured using steady-state method in accordance with ISO 8302. The instrument is shown in Figure 3. The steady-state method is based on the principle of the heat transfer balance, that is, the heat transfer rate is consistent with the heat dissipation rate [22]. If the temperature gradient at both ends of the material is measured and the heat flow through its unit area is known, the value of the thermal conductivity can be calculated through the Fourier formula shown in Equation (1).

(1) $$\lambda_{m}=\frac{q\delta }{\Delta T}$$

where, $\lambda_{m}$—thermal conductivity coefficient, W/(m. K), *q*—constant heat flux on one side of the heating plate, W/m^2^, ΔT—temperature difference between cold plate and hot plate, K.

### 2.3. Combustion Tests

The combustion performance of CEPS was measured by two parameters according to the standard GB 8624, including heat of combustion and fire growth rate index (FIGRA).

Gross heat of combustion (PCS) was calculated by Equation (2) according to the standard GB/T 14402.

(2) $$PCS=\frac{E\times \left(T_{m}-T_{i}+c\right)-b}{m}$$

where PCS is gross heat of combustion (MJ/kg), E is the water equivalent other than water in calorimetric system (MJ/K), *T_i_* and *T_m_* is the starting and maximum temperature (K), respectively, *b* is the correction value of combustion calorific value of combustion supporting materials used in the test (MJ), *c* is temperature correction value of external heat exchange (K), and *m* is the weight of specimens (kg).

The fire growth rate index (FIGRA) is calculated according to the GB/T 20284. FIGRA is the maximum value of the heat release rate (HRR) of the sample combustion to its corresponding time, which is used to classify combustion performance. The larger the FIGRA, the easier the material is to burn; the faster the fire grows, the higher the fire risk coefficient. The FIGRA_0.2MJ_ is the combustion growth rate index when the heat released from the combustion of the sample reaches 0.2 MJ. THR_600s_ is the total heat release of the specimen in the first 600 s (300 s ≤ t ≤ 900 s) after fired by the main burner.

The butane combustion test is an intuitive qualitative index to evaluate the flame retardance of exterior wall insulation materials [4]. In this paper, CEPS specimens were directly exposed to butane flame with temperature up to 1500 °C for 1 h, the combustion process was recorded and the difference of residues after combustion was compared.

### 2.4. Scanning Electron Microscopy (SEM) Tests

In order to further investigate the interfacial transition zone (ITZ) of CEPS, SEM was employed. FEI Quanta 250 FEG (FEI, Hillsboro, OR, USA) apparatus was used for capturing the SEM images. The microstructures of the CEPS specimens were analyzed by SEM images.

## 3. Results and Discussion

### 3.1. Physical and Mechanical Properties

**Figure 4:**
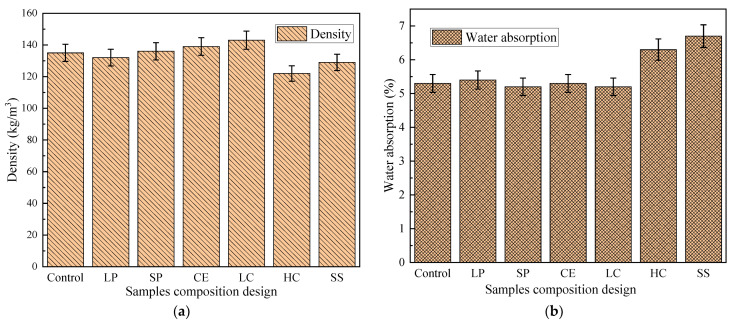

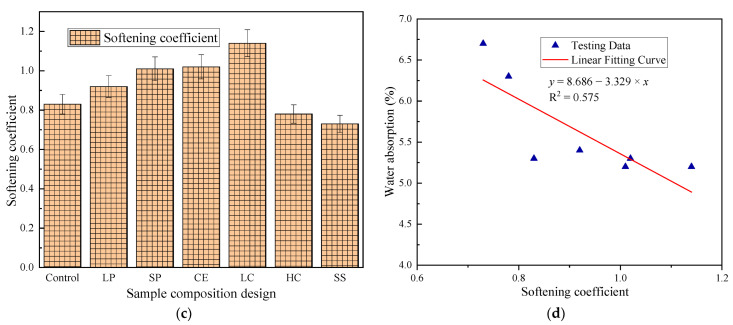
The physical properties of CEPS with different compositions: (**a**) density, (**b**) water absorption, (**c**) softening coefficient, and (**d**) the relationship between softening coefficient and water absorption.

In this study, the main purpose is to meet the requirements of mixture density between 120 and 140 kg/m^3^; the compressive strength, tensile strength, and flexural strength greater than 0.15 MPa, 0.1 MPa, and 0.2 MPa, respectively. The tensile strength ratio and softening coefficient equal or greater than 75% and 0.7, respectively; water absorption equal or less than 10%. Figure 4a presents the density results of different CEPS. As it can be seen, cement-based binder affects the density of mixtures the most. The density of single particle size-EPS (SS) is small. At the same compression ratio, the particle size of EPS has no effect on the density. The results have also been acknowledged by other researchers [11,23]. The water absorption test data of the seven CEPS groups are plotted in Figure 4b. It can be seen that the water absorption of SS, LC and LP are relatively large. In terms of the single particle size and high content of EPS particles, large voids in the structure mean high water absorption [24]. The water absorption of EPS with a large particle size is larger than that of EPS with a small particle size, and the particle size of larger diameter EPS cause larger voids. The softening coefficient test results are shown in Figure 4c. The greater the softening coefficient, the less the material is affected by the external environment, and the better the freeze-thaw resistance and aging resistance. The softening coefficient is most affected by cement-based binder. Sufficient content and reasonable distribution of cement-based binder are the key to improving the softening coefficient of CEPS. When EPS particle size is small and cement-based binder is evenly distributed, the softening coefficient will be higher after EPS is coated. The relationship between softening coefficient and water absorption is analyzed, and the results are shown in Figure 4d. The data show that the correlation between the two is not strong. High water absorption of the material does not mean low softening coefficient, and vice versa. That is because the content of cement-based binder and its distribution in CEPS are the key factors affecting the softening coefficient.

**Figure 5:**
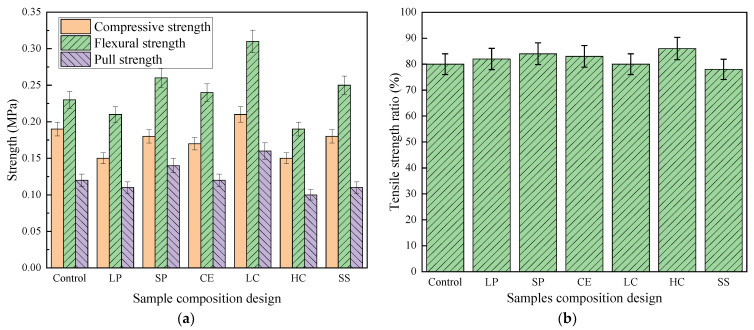
The strength and residual tensile strength ratio of seven CEPS groups: (**a**) strength and (**b**) tensile strength ratio.

The mechanical test results of CEPS are shown in Figure 5. As illustrated in Figure 5a, the compressive strength and flexural strength of low EPS content are the highest, followed by the small particle size of high EPS content. With the increase of particle size, the compressive and tensile strength decrease. The compressive strength and tensile strength of the larger EPS particle size are lower than that of the higher EPS content [25]. There is a similar trend in the pull strength test results. It shows that the performance of cement-based binder is the primary factor affecting the strength.

As shown in Figure 5b, the influence of the freeze-thaw cycle on the mixture: tensile strength ratio (TSR) with lower EPS content is the highest, followed by small particle size with higher EPS content. In terms of the high elasticity of EPS, the volume required for ice expansion after water enters the gap of CEPS is achieved by compressing EPS, so the freeze-thaw strength is significantly higher than that of ordinary concrete.

### 3.2. Thermal Conductivity

The lower the thermal conductivity of the external wall insulation material, the less likely the heat is to penetrate the wall, and the better the energy-saving effect of the building. In general, the thermal conductivity of EPS particles is 0.039 W/(m. K) [26]. To make the thermal conductivity of the EPS mixture as close as possible to 0.039 W/(m. K), the cement-based binder should be added as less as possible. On the contrary, with less cement-based binder, the flame retardant effect will become worse [27]. In previous studies, the thermal conductivity of organic materials is generally about 0.039, even lower than 0.039 W/(m. K) after adding aerogel gel materials (high costed but low strengthened material), while the thermal conductivity of inorganic insulation materials is generally greater than 0.07 W/(m. K), as shown in the literature [1,24,28].

**Figure 6:**
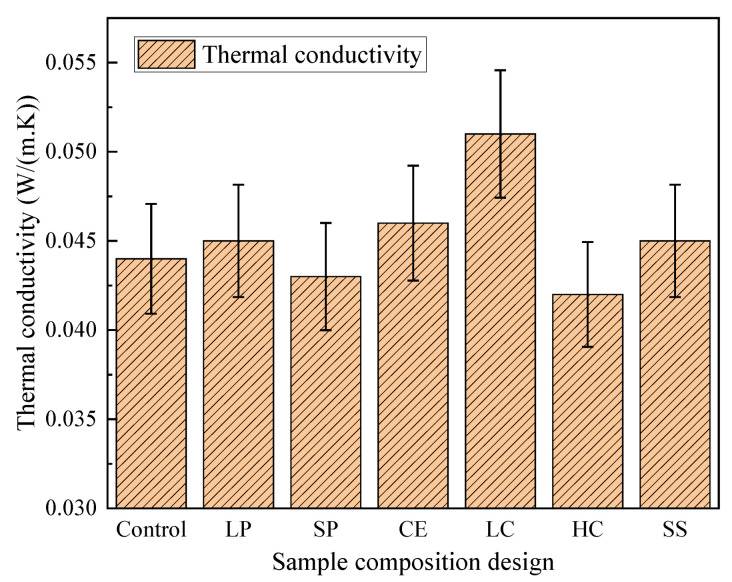
The results of thermal conductivity.

The target thermal conductivity of the mixture designed in this paper is less than or equal to 0.045 W/(m. K). The test results of thermal conductivity are presented in Figure 6. The thermal conductivity with higher EPS content is the lowest, followed by that with higher EPS content but smaller particle. With the decrease of particle size, the thermal conductivity decreases, the thermal conductivity with a certain gradation is better. In the unit volume of CEPS, the number of small particles is larger than large particles, and the compact packing density is larger. That is, during the heat transfer process, the pore interface between the pores of CEPS cement-based binder and EPS particles increases, so the heat conduction path is extended, which slows down the heat conduction rate of CEPS, and finally reduces the thermal conductivity.

### 3.3. Combustion Behavior

The combustion characteristics of the samples include the heat release rate HRR, combustion value, total heat release within 600 s-THR_600s_, and the combustion growth rate index FIGRA_0.2MJ_. HRR represents the speed and size of heat released by the fire source, and it also reflects the ability of the fire source to release heat. The greater the HRR, the more heat the combustion feedback gives to the surface of the material, resulting in the accelerated pyrolysis speed of the material and the increase in the production of volatile combustibles, thus accelerating the spread of the fire. Generally, the HRR curve is divided into three stages: the initial growth stage, full development stage and weakening stage [29]. The combustion value of building materials is an important parameter to characterize the potential fire risk of building materials, and it is the essential basic data to calculate the heat released by the combustion of building materials and the fire load. It can be used to evaluate the potential fire risk of building materials products. The other two characteristics have been shown in Section 2.3.

**Figure 7:**
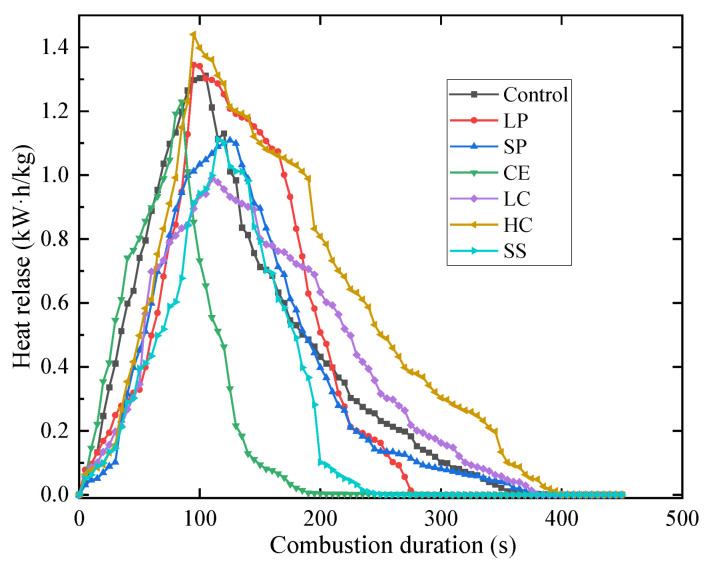
The results of HRR.

The HRR of the seven CEPS are shown in Figure 7. Similar to other exterior wall insulation materials, the CEPS, in the initial growth stage, will release a large amount of heat rapidly; but in the second stage, the full development stage the release will not maintain for a long time but directly weaken. The reasons for the performance are that EPS particles are wrapped by cement-based binder, and that the volume content is lower than that of other materials. The HRR of the sample with higher EPS content and larger particles is the highest, followed by the one with higher EPS content but smaller particles. With the increase of particle size, the combustion performance becomes worse, and the HRR of the sample with larger particles is lower than that of higher EPS content. The HRR of the CE is lower than that of any other materials because the coating material prevents the combustion, so the combustion performance is significantly improved. The finding is consistent with previous studies in which silica-coated EPS improved flame retardance, smoke suppression and mechanical strength [17].

**Figure 8:**
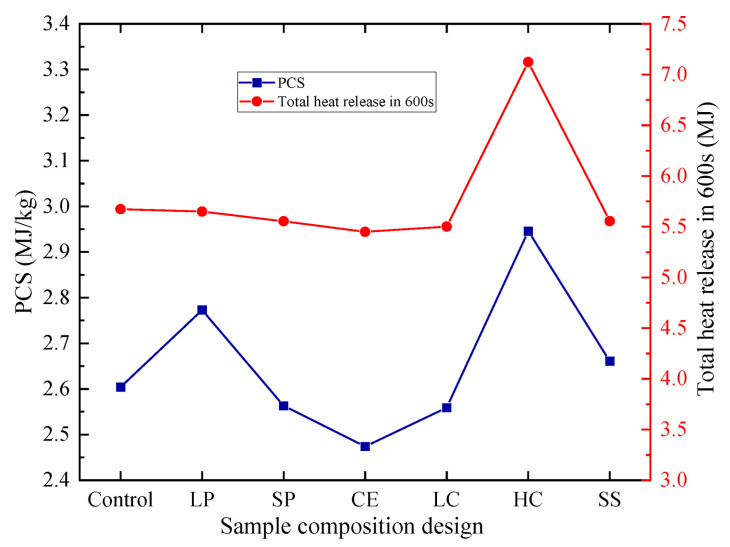
The gross heat of combustion and THR_600_ of seven CEPS groups.

As described in Section 2.3, the combustion value is calculated by PCS. The mixture target PCS designed in this paper is less than or equal to 3.0 MJ/kg. The combustion value and THR_600_ test results are shown in Figure 8. The content of EPS is the main factor affecting PCs and THR_600s_. If the content is larger, both are larger. The second factor is the size EPS particles. When the particle size is larger, CEPS with the same volume has less interface and is easier to burn.

**Figure 9:**
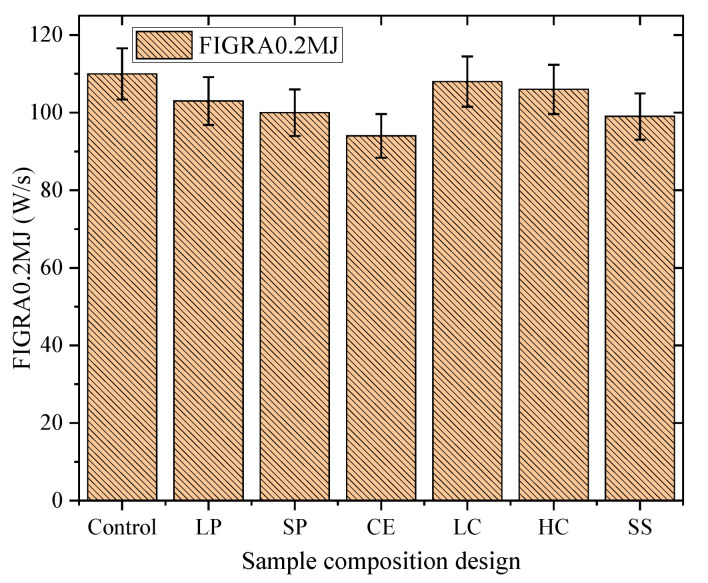
The results of FIGRA_0.2MJ._

The target FIGRA_0.2MJ_ of the mixture designed in this paper is less than or equal to 120 W/s. The result of FIGRA_0.2MJ_ is shown in Figure 9. As is shown, FIGRA_0.2MJ_ of the sample with larger particles needs to be strictly controlled. In addition, the nano coating can be used to reduce the value of FIGRA_0.2MJ_ to a certain extent.

**Figure 10:**
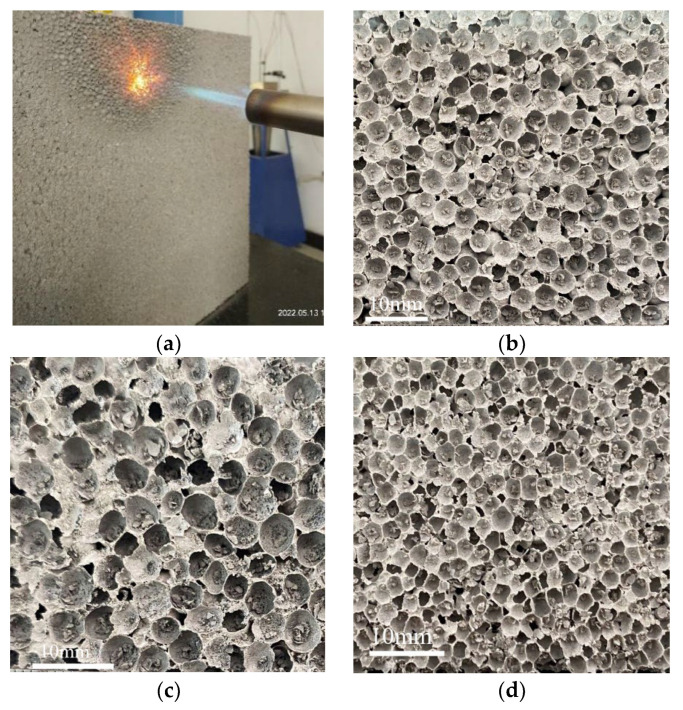

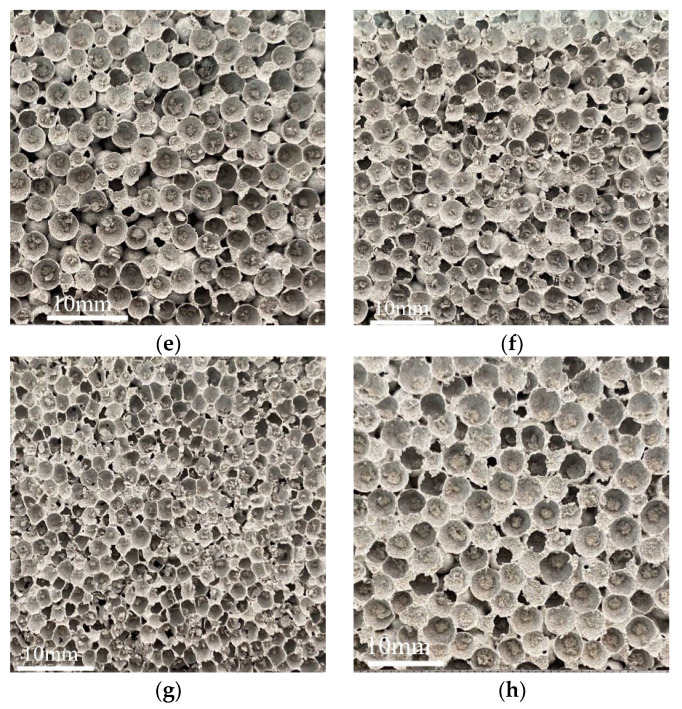
The residue after burning test: (**a**) specimens under test, (**b**) control, (**c**) LP, (**d**) SP, (**e**) CE, (**f**) LC, (**g**) HC, and (**h**) SS.

After being exposed in high temperature, EPS is melted, and the results of residue are shown in Figure 10. It can be seen that the residues of all test pieces are loose and honeycomb. As EPS is dispersed in the cement-based binder, no drops are produced during the combustion process, and the shape is not collapsed and deformed, which meets the requirements of grade A2 flame retardance. The size and content of EPS particles are different, showing different structural states. Figure 10d, g show that the residues mainly composed of inorganic substances present dense and uniform morphology and fewer voids, which can effectively prevent heat and mass transfer between the flame zone and the condensing phase. It results in the reduction of HRR, PCS, THR_600s_ and FIGRA_0.2MJ_, and better flame retardance. Figure 10c,f,h show similar contour characteristics, except that EPS particle sizes and compositions are different; the flame retardance mechanism is similar, so there is no difference in its flame retardance performance. Figure 10e shows that the combustion residue of the sample with EPS coated is not significantly different from that of other samples, but the combustion HRR, THR_600s_ and FIGRA_0.2MJ_ are lower, which can provide a precious opportunity for personnel to escape and materials to be rescued in case of fire. In addition, there is no shape collapse after combustion, which is closely related to the addition of nitride, aluminum oxide, etc. in the cement-based binder. The addition of these flame-retardant materials prevents the open fire from further hidden combustion.

### 3.4. Microstructure Analysis

**Figure 11:**
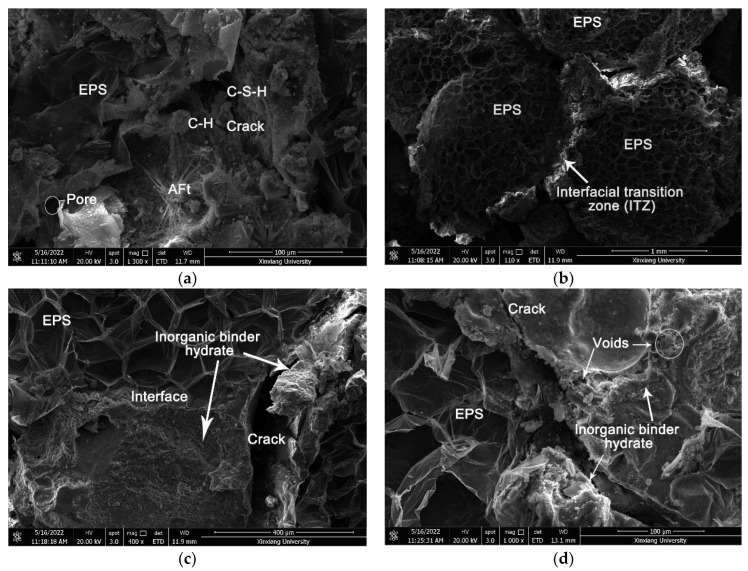
The microstructures of the CEPS: (**a**) the cement-based binder after hydration, (**b**) ITZ, (**c**) crack, and (**d**) voids and crack.

The microstructures of the CEPS are analyzed by SEM imaging to reveal the mechanisms of modification. The cement-based binder plays an important role in the bonding of EPS. If it is not sufficiently adhesive, it will lead to strength damage, freeze-thaw damage, etc., especially in the interface area between EPS and cement-based binder. Thus, this study focuses on the interface region of binary materials and the internal structure of cement-based binder. The microstructure images are shown in Figure 11a–d. Figure 11a shows the microstructure of the cement-based binder after hydration. It can be seen from the image that the cement-based binder is partially hydrated, which is composed of hydrated calcium silicate (C-S-H) gel, calcium hydroxide (C-H) and Ettringite (AFT) crystals, as well as some voids and cracks. The cement-based binder first forms the crystal nucleus of the hydration product in the hydration process [30]. The length of the crystal nucleus means that the hydration product grows up and adheres to each other in a staggered way. After curing, the hydration product develops, grows, condenses, hardens, and further solidifies to attain the required strength. In a word, cement-based binder is the skeleton to obtain mechanical strength.

As illustrated in Figure 11b, the EPS content of the sample is high, while the content of cement-based binder is low. When it is cut into the specimen, bond failure occurs, and the failure interface is located between EPS particles and cement-based binder. As shown in Figure 11c, the cement-based binder of the sample is relatively sufficient, the interface zones significantly improved. The cement-based binder is evenly distributed on the surface of EPS particles, making the interface adhesion tighter, so the composite strength of CEPS is higher. It can be observed that many EPS particles break at the damaged interface rather than at the interface zones. Figure 11d reveals the microstructure of the CEPS with nano materials coated on the surface of EPS. Compared with other groups of CEPS, CEPS coated with nano materials on the surface of EPS has better interface adhesion, more voids after hydration, more organized speed of heat transmission, and lower thermal conductivity. Therefore, this porous structure has great advantages for improving flame retardance.

### 3.5. Statistical Analysis

In order to statistically study the contribution of EPS particle size and content to the strength, thermal conductivity and flame retardance of CEPS, the influence of significant difference analysis is used. Table 2 summarizes the statistical results of strength, thermal conductivity, and flame retardance of different EPS compositions. The three letters H, M and N are used to represent high significant difference (*p* < 0.01), medium significant difference (0.01 < *p* < 0.05) and no significant difference (*p* > 0.05).

The statistical results in Table 2 show that the content of EPS has a significant impact on the thermal conductivity and flame retardance, and the corresponding cement-based binder content has at least a moderate impact on the mechanical and physical properties. The flame retardancy of EPS coated with nanoparticles is significantly affected, while others are slightly affected. The effect of EPS particle size on TSR is significant, while the effect on temperature and flame retardance is medium.

### 3.6. Cost Analysis

Table 3 lists the unit prices of EPS and cement-based materials purchased in the Chinese market in June 2022, and the cost of production of per cubic meter CEPS. The unit price is 30% lower than that of rock wool insulation board, but the performance is improved many times.

## 4. Conclusions

In this study, the effect of EPS particle composition, content and surface-coated on characteristics of incombustible thermal insulation mixture have been investigated. The main conclusions are summarized as follows:

(1) The content of EPS particles has the greatest influence on the mechanical properties, thermal conductivity and flame retardancy of the designed mixture.

(2) The mixture made of smaller EPS particles has higher mechanical strength; cement-based slurry forms honeycomb isolation effect, and so FIGRA_0.2MJ_ is lower when burning.

(3) Although the surface coating reduces the physical and mechanical properties of the mixture and has little effect on the thermal conductivity, it delays the heat and smoke release.

(4) The mechanical performance of the CEPS is significantly affected by the materials composition and adhesive efficiency of the interfaces. This is consistent with the SEM observations of the microstructure of the EPS interface and cement-base slurry.

The future work could be directed toward how to integrate external wall insulation and decorative materials, reduce the difficulty of construction, and improve construction efficiency.

## Figures and Tables

**Table 1:** The description of the prepared CEPS mixture.

Mix ID	Particle Size of EPS (mm)	The EPS Dosage (m^3^/m^3^)	Cementing Materials (kg/m^3^)	W/C Ratio
Control	3–4	1.7	70	0.45
Large particle-CEPS (LP)	4–6	1.7	70	0.45
Small particle-CEPS (SP)	2–3	1.7	70	0.45
Coated-EPS-CEPS (CE)	3–4	1.7	70	0.45
Low- EPS content-CEPS (LC)	3–4	1.4	80	0.45
High- EPS content-CEPS (HC)	3–4	2.0	60	0.45
Single particle size-EPS-CEPS (SS)	3	1.7	70	0.45

**Table 2:** A significant analysis of strength, thermal conductivity, and flame retardance of CEPS.

	Physical and Strength Properties				Thermal Conductivity	Flame Retardance		
	Compressive Strength	Flexural Strength	Softening Coefficient	TSR		HRR	THR_600s_	FIGRA_0.2MJ_
EPS content effect								
LC	H	H	M	H	H	H	H	H
HC	H	M	M	H	H	H	H	H
EPS Particle size effect								
LP	M	N	H	H	M	M	M	M
SP	N	M	H	H	M	M	M	M
SS	N	N	M	H	M	M	M	M
EPS coated effect								
CE	N	N	M	N	M	H	H	H

Note: H, M and N are used to represent high significant difference (*p* < 0.01), medium significant difference (0.01 < *p* < 0.05), and no significant difference (*p* > 0.05).

**Table 3:** The average unit cost of CEPS.

Mix ID	EPS (RMB/m^3^)	Cementing Materials (RMB/tonne)	Total Price (RMB/m^3^)
rock wool	-	-	450
Control	90	1200	297
LP	95	1200	324.5
SP	100	1200	340
CE	110	1200	331
LC	90	1200	327
HC	90	1200	300
SS	100	1200	314

## Data Availability

Data is contained within the article.
